# Supplementary figures and images for: Evaluation of Three Serological Tests for Diagnosis of Canine Brucellosis
Source: Microorganisms. 2023 Aug 26;11(9):2162. doi: 10.3390/microorganisms11092162 (PMC10536495; doi:10.3390/microorganisms11092162)

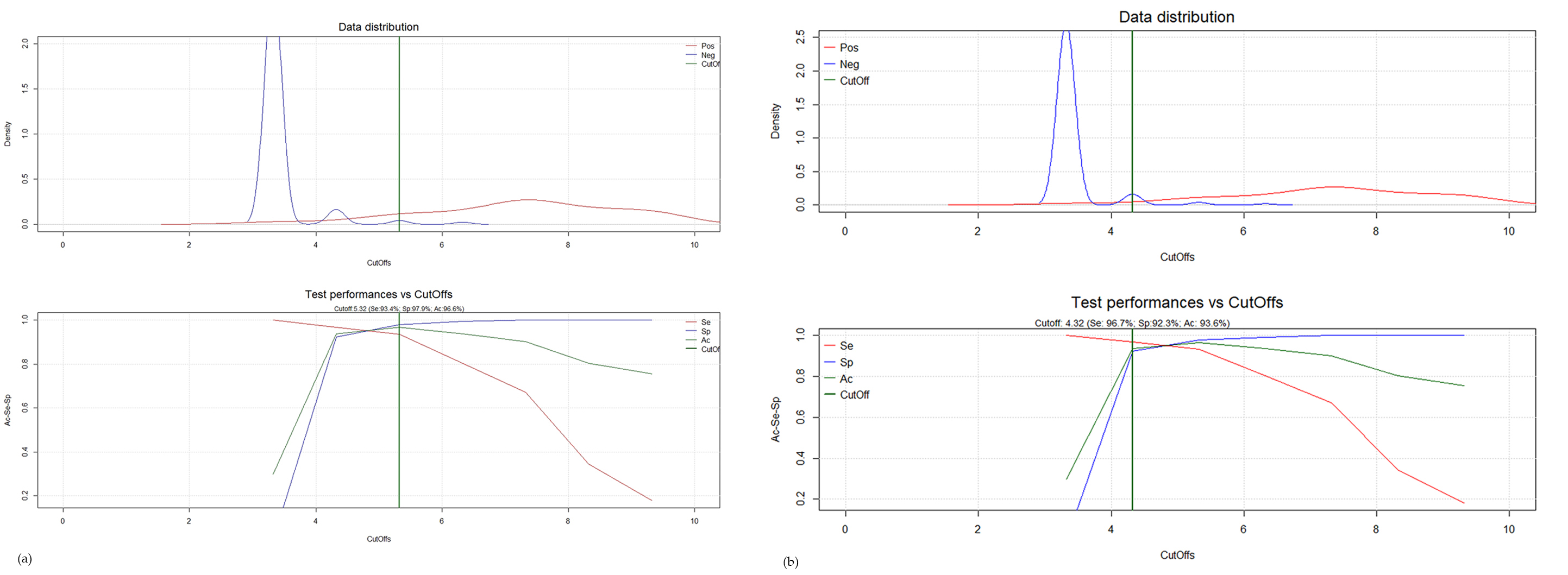

Supplement: Supplementary file 1 [file microorganisms-11-02162-s001.zip › S1a_b.jpg]

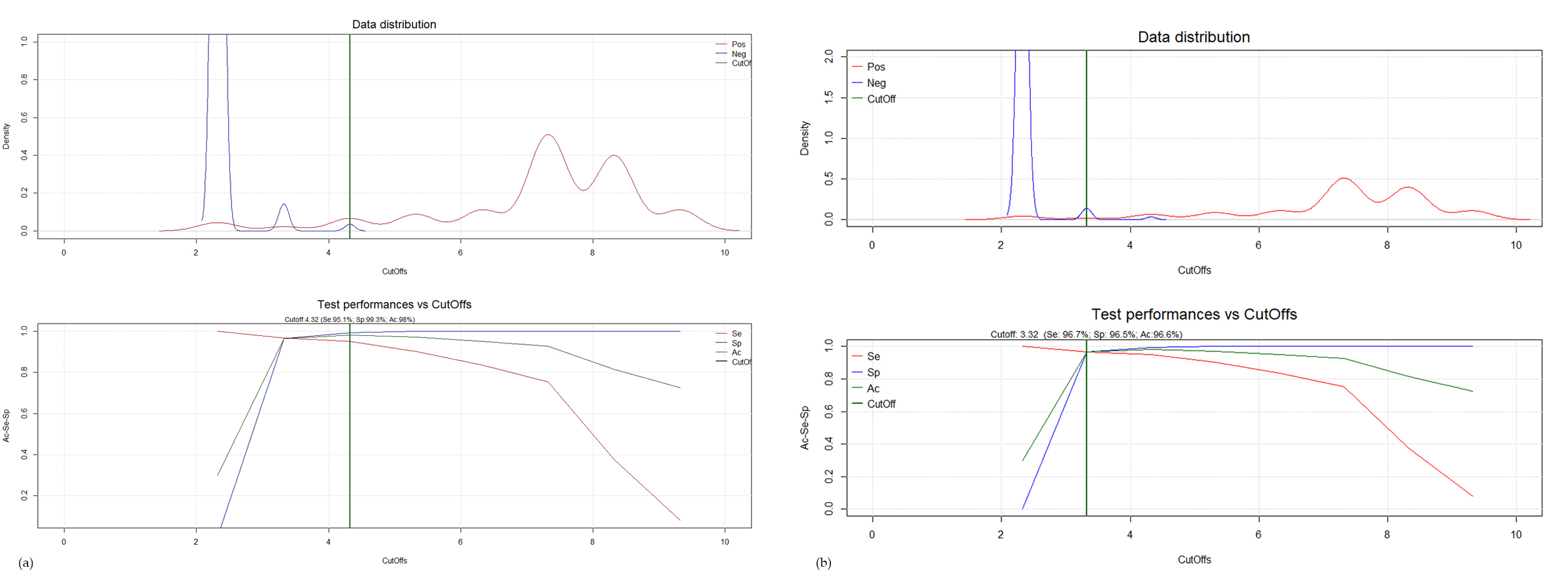

Supplement: Supplementary file 1 [file microorganisms-11-02162-s001.zip › S2a_b.jpg]

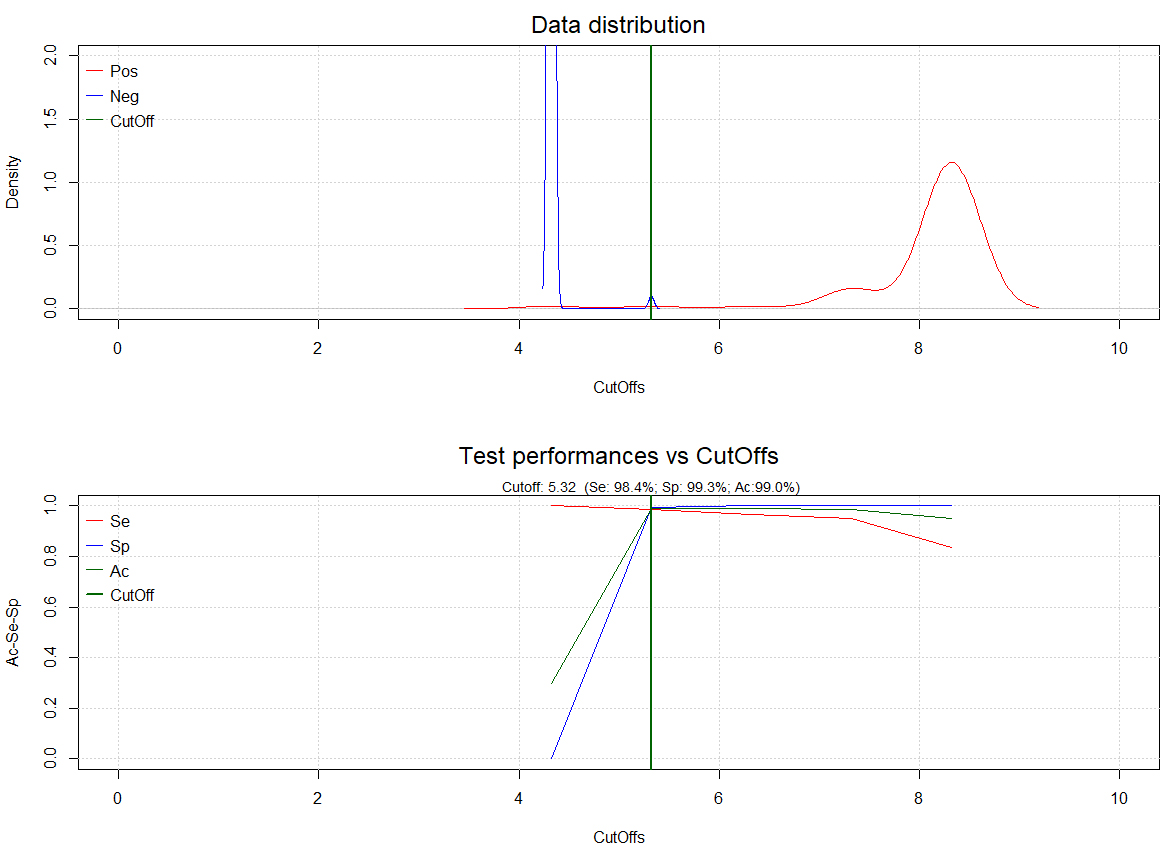

Supplement: Supplementary file 1 [file microorganisms-11-02162-s001.zip › S3.jpg]
